# Adherence to a Healthy Beverage Score Is Associated with Lower Frailty Risk in Older Adults

**DOI:** 10.3390/nu14183861

**Published:** 2022-09-18

**Authors:** Ligia J. Dominguez, Carolina Donat-Vargas, José R. Banegas, Mario Barbagallo, Fernando Rodríguez-Artalejo, Pilar Guallar-Castillón

**Affiliations:** 1 Faculty of Medicine and Surgery, “Kore” University of Enna, 94100 Enna, Italy; 2 Geriatric Unit, Department of Internal Medicine and Geriatrics, University of Palermo, 90100 Palermo, Italy; 3 Department of Preventive Medicine and Public Health, School of Medicine, Universidad Autónoma de Madrid-IdiPaz, CIBERESP (CIBER of Epidemiology and Public Health), 28029 Madrid, Spain; 4 IMDEA-Food Institute, CEI UAM + CSIC, 28049 Madrid, Spain; 5 Unit of Nutritional Epidemiology, Institute of Environmental Medicine, Karolinska Institutet, 17177 Stockholm, Sweden

**Keywords:** aging, frailty, healthy beverages, prospective cohort, ENRICA study

## Abstract

Many beverages include bioactive components and energy but are frequently not considered in diet quality estimations. We examined the association of a healthy beverage score (HBS) with incident frailty in older adults from the Seniors-ENRICA-1 cohort. We used data from 1900 participants (mean ± SD age 68.7 ± 6.4 years, 51.7% women), recruited in 2008–2010 and followed-up until 2012 assessing food consumption at baseline with a validated diet history. The HBS was higher for increasing consumption of low fat milk, tea/coffee, lower consumption of whole milk, fruit juice, artificially sweetened beverages, sugar-sweetened beverages, and moderate intake of alcohol. Frailty was considered as having ≥3 criteria: exhaustion, low-physical activity, slow gait speed, weakness, and weight loss. We performed logistic regression analyses adjusted for potential confounders. During a 3.5 y mean follow-up, 136 new cases of frailty occurred. Compared to the lowest sex-specific HBS tertile, the fully adjusted odds ratio (95% confidence interval) of frailty was 0.59 (0.38, 0.92) in the intermediate tertile, and 0.52 (0.31, 0.88) in the highest tertile, *p* trend = 0.007. Results for slow gait speed were 0.79 (0.58, 1.07) and 0.71 (0.51–0.99), *p* trend = 0.033. Therefore, adherence to HBS was inversely associated with incident frailty and slow gait speed. HBS can help on the beverage quality evaluation, highlighting beverage importance as contributors to diet and to health.

## 1. Introduction

The proportion of older people in the population is increasing in every country. In those with a high life expectancy, such as in Spain, a person retiring can still expect to live more than 20 years [1,2]. Even more, demographic data predict that half of the children alive in 2010 in regions with the highest life expectancy will be centenarians [3]. However, this optimistic scenario might be overshadowed because aging may be accompanied by multiple chronic conditions including frailty, which is a prelude to disability. The current prevalence of frailty among older adults is estimated to be 12–24% [4].

Frailty is a broad concept that can be viewed as “a syndrome of geriatric syndromes” resulting from multiple causes and characterized by decreased endurance and strength, and reduced physiological function, which in turn increases vulnerability to even minor stressors. Thus, it confers a double to triple risk of multi-morbidity, disability, institutionalization, hospitalization, and mortality [5,6,7]. Owing to the elevated prevalence of this syndrome, and the fact that frailty is potentially reversible, there is growing interest in identifying risk factors for frailty as well as possible interventions to avoid or delay its onset [8].

Food consumption is a crucial determinant of frailty, and quantitative as well as qualitative adequate dietary intakes are key modifiable risk factors for this syndrome. To estimate frailty, the most widely used operational definition is that provided by Fried et al. based on the frailty phenotype [9]. As such, poor nutritional status may potentially affect the five criteria used in this frailty phenotype, including exhaustion, low physical activity, slow gait speed, weak grip strength, as well as unintentional weight loss [10]. A recent meta-analysis including fifteen cohort and cross-sectional studies reported that a greater adherence to a healthy dietary pattern (mainly based on solid foods) was associated with a notable lower risk of frailty [11].

Beverages are essential to maintain hydric balance, many of them contain nutrients, bioactive components, as well as energy, and they can interact with other constituents of the diet affecting health. Even so, only a single or few beverages are included in the most commonly used dietary indexes. For example: dairy (comprising milk, yogurt, and cheese together) and wine are included in the Mediterranean diet indexes [12]; dairy (also comprising milk and milk products) is included in the Dietary Approaches to Stop Hypertension (DASH) index [13]; dairy and added sugars (comprising sugar-sweetened beverages (SSBs)) are included in the Healthy Eating Index (HEI)-2015 [14]; carbonated and/or SSBs, as well as wine, are considered in the energy-restricted Mediterranean Diet Screener (er-MEDAS) [15]. However, other beverages that are generally consumed daily such as tea, coffee, and artificially sweetened beverages (ASBs) are not usually included in *a priori* dietary indexes. As a result, beverages are not fully considered when estimating the quality of diet.

Likewise, although drinking beverages such as milk [16], coffee [17], and SSBs [18] have already been studied in relation to frailty risk, the role of a healthy beverage index including all types of beverages has not been explored. Therefore, we aimed to examine the association of a healthy beverage score (HBS) with incident frailty in older adults.

## 2. Materials and Methods

### 2.1. Study Design and Participants

We used data from participants in the Seniors-ENRICA-1 cohort. In brief, the ENRICA Study was conducted with 13,105 individuals aged 18 years or older, who were selected between June 2008 and October 2010 by random stratified cluster sampling of the non-institutionalized Spanish population. Those who were 60 or older at baseline and those who were followed-up until 2012 to update data collection constituted the Seniors-ENRICA-1 cohort (*n* = 2614). At baseline and at follow-up, trained personnel collected information in three stages: (1) a telephone interview to obtain data on sociodemographic factors, health behaviors, morbidity, and healthcare services use; (2) a first home visit to collect blood and urine samples; and (3) a second home visit to perform a physical examination and to obtain habitual food consumption with a computerized dietary history. Detailed methods of the Seniors-ENRICA-1 cohort have been reported elsewhere [19,20].

From the total participants in the cohort, 95 died during follow-up. Among the 2519 participants who were alive, we excluded 209 who had frailty at baseline or lacking data on frailty at baseline; 16 who did not have complete diet information or had an implausibly high- or low-energy intake (outside the range of 400–3500 kcal/day for women and 600–4200 kcal/day for men), and 394 with no information on frailty at the end of follow-up. Thus, the analytical sample included 1900 participants.

The study protocol was approved by the Clinical Research Ethics Committee of the *La Paz* University Hospital in Madrid (Project identification code: HULP PI-1793). All participants provided written informed consent.

### 2.2. Dietary Assessment

At baseline, the habitual food consumption was assessed with a validated computerized face-to-face dietary history (DH-ENRICA) developed from that used in the European Prospective Investigation into Cancer and Nutrition (EPIC) cohort study in Spain [21]. Food consumption during a typical week, representative of dietary consumption in the previous year, was reported by the participants; DH-ENRICA allowed to register over 860 foods and dishes from the Spanish tradition; a set of 120 pictures was utilized in order to help estimating the portion sizes (g/day) of each food/dish that was consumed. Nutrients and energy intake were calculated with standard food composition tables [22,23]. Study participants reported all beverages they had consumed at least once every two weeks.

### 2.3. Healthy Beverage Score

We selected a score of seven components previously reported in a cohort of patients with chronic renal insufficiency [24], using a modification from the original Health Beverage Index (HBI) proposed by Duffey and Davy [25] and based on recommendations from the Healthy Beverage Guidance System [26]. To assess overall beverage quality, we adapted the HBS to the regular beverage consumption of older Spanish adults. The HBS ranged from 7 to 28 points (the higher the index, the better the beverage quality). The seven components were classified into adequacy components, which represented beverages that scored positively (low fat milk, and tea/coffee), and moderation components for beverages that scored negatively (whole milk, fruit juice, ASBs, SSBs, and alcohol). The scoring for each component ranged from 0 to 4 and was based on consumption; thus, for low-fat milk as well as for tea/coffee, the scoring was based on quartiles; for whole milk, it was based on tertiles among consumers; for fruit juice, ASBs, and SSBs (which have a much lower consumption in this population), any consumption was considered as detrimental (1 point), so that 4 points corresponded to no consumption; finally, for alcohol, a moderate intake was considered as beneficial (4 points), and no consumption or heavy drinking as detrimental (1 points); the threshold for moderate to heavy alcohol drinking was considered at 24 g/day for women and 40 g/day for men [27] (Table 1). A total of 99 different beverages were included in the HBS (Supplemental Table S1).

### 2.4. Ascertainment of Frailty

We used the frailty definition proposed by Fried et al. in the Cardiovascular Health Study [9]. In particular, frailty was defined as having three or more of the five phenotypic criteria proposed by Fried et al.: (1) exhaustion, considered when participants answered “yes, 3 to 4 or more days a week” to at least one of two questions from the Center for Epidemiologic Studies-Depression Scale: “I felt that anything I did was a big effort” or “I felt that I could not keep on doing things” [28]; (2) muscle weakness, when maximum grip strength on the dominant hand in two consecutive measurements using a Jamar dynamometer and adjusted for sex and body mass index (BMI) was in the lowest quintile for our cohort [29,30]; (3) low physical activity, defined as walking ≤ 2 h/week in women or ≤2.5 h/week in men; (4) slow gait speed, considered as the lowest cohort-specific quintile in a 3 m walking speed test, adjusted for sex and height [29,31]; and (5) unintentional weight loss, when ≥4.5 kg of body weight was lost in the previous year.

### 2.5. Other Variables

Other variables assessed at baseline included socio-demographic factors (age, sex, educational level), and tobacco consumption. Weight and height were measured at home under standardized conditions, and the body mass index (BMI) was calculated as weight (kg) divided by square height (m^2^). The validated EPIC-Spain cohort questionnaire was used to collect information on physical activity [32]. Hypertriglyceridemia was considered for fasting serum triglycerides ≥150 mg/dL; hypercholesterolemia was defined as fasting serum total cholesterol level ≥200 mg/dL or when the participant was taking lipid-lowering medications; hypertension was considered for blood pressure ≥140/90 mmHg or taking antihypertensive medication. The number of chronic conditions included any of the following physician-diagnosed conditions reported by the participants: coronary heart disease, stroke, chronic respiratory disease, cancer at any site, osteoarthritis/arthritis, diabetes, and depression requiring treatment. The number of medications used was checked against the drug packages by a nurse. Adherence to the Mediterranean dietary pattern was assessed with the index described by Trichopoulou without considering alcohol consumption (range 1–8) [12]. In this study, total energy intake (kcal/day) was used to adjust for covariates. Because total energy intake (kcal/day) is influenced by body size, we also used the total energy intake per ideal body weight (kcal/ideal body weight/day) as a covariate [33].

### 2.6. Statistical Analyses

Participants were classified into sex-specific tertiles of their HBS scores. The association between HBS and the risk of frailty were summarized as odds ratios (ORs) and their 95% confidence interval (CI), obtained from non-conditional multivariable logistic regressions taking the lowest HBS tertile as the reference. We also tested the association of HBS with each frailty criterion. Socio-demographic, clinical variables, and lifestyle parameters associated with frailty according to the medical literature were considered as potential confounders. Progressive levels of adjustments were included to build three logistic models. Model 1 was adjusted for age (years) and sex; Model 2 was additionally adjusted for educational level (no formal education, primary, and secondary or higher), smoking status (no smoker, former, and current smoker), BMI (<25, ≥25 to <30, and ≥30 kg/m^2^), physical activity (inactive, moderately inactive, moderately active, and active), total energy intake (kcal/day), fiber intake (g/day), fruit consumption (g/day), vegetables consumption (g/day), hypertriglyceridemia (yes/no), hypercholesterolemia (yes/no), high blood pressure (yes/no), number of self-reported chronic conditions (0, 1, and ≥2), and number of medications (≤3 and >3); Model 3 was additionally adjusted for adherence to the Mediterranean diet [12] (excluding alcohol to avoid over-adjustment, maximum score = 8) and excluding fruit, and vegetable consumption. We used stochastic regression for the imputation of missing values (<1%) in covariates (BMI, hypertriglyceridemia, hypercholesterolemia, and high blood pressure). All results were checked against models built after selecting participants with complete information for all covariates. As sensitivity analyses and to ensure the robustness of the results, we also performed the analyses across quartiles of HBS adherence, among robust participants at baseline (without any frailty criteria), and after controlling for frailty criteria at baseline. Since many older adults should not consume alcohol (e.g., because they take medications that interact with it or have pathologies that are aggravated by alcohol), while they should know that if they follow the HBS their health can improve even if they do not drink alcohol, we also performed the analyses excluding alcohol consumption and adjusting the model for alcohol. All *p* values were 2-tailed, and significance was set at *p* < 0.05. We performed the analyses with Stata/SE, version 16 (Stata-Corp, College Station, TX, USA).

## 3. Results

The mean age of the participants was 68.7 ± 6.4, and 51.7% were women. Participants in the highest tertile of HBS smoke more frequently and had less energy intake compared with those in the lowest tertile (Table 2).

After a mean follow-up of 3.5 years, 136 (7.2%) new cases of frailty occurred. There was an inverse significant association when comparing extreme tertiles of the HBS in all models. Even after adjusting for the Mediterranean dietary pattern, the results remained significant. In Model 3, the ORs (95% CI) of frailty risk across tertiles of HBS were: lowest tertile 1 (ref.), intermediate tertile 0.59 (0.38, 0.92), and highest tertile 0.52 (0.31, 0.88); *p* for linear trend 0.007 (Table 3).

When considering the risk of incident frailty according to quartiles of adherence to HBS, the results were similar (Supplemental Table S2). Likewise, the results were similar among robust participants at baseline (without any frailty criteria) (Supplemental Table S3), after controlling for frailty criteria at baseline (Supplemental Table S4), and excluding alcohol consumption from the HBS and adjusting the model for alcohol consumption (Supplemental Table S5). When we used total energy intake per ideal body weight (kcal/ideal body weight/day) as a covariate, the results were similar and remained significant (Supplemental Table S6). Odds ratios of covariates are shown in Supplemental Table S7.

Each frailty criterion was also analyzed separately. During follow-up, there were 255 (13.4%) participants who self-reported exhaustion, 656 (34.5%) with weak grip strength, 302 (15.9%) with low physical activity, 280 (14.7%) with slow gait speed, and 150 (7.8%) with unintentional weight loss in participants who did not report these criteria at baseline. When comparing extreme tertiles, HBS was inversely associated with each frailty criterion, although statistical significance was achieved for slow gait speed. In Model 3, the ORs (95% CI) for slow gait speed risk across tertiles of HBS were: lowest tertile 1 (ref.), intermediate tertile 0.79 (0.58, 1.07), and highest tertile 0.71 (0.51, 0.99); *p* for linear trend 0.033 (Table 4).

## 4. Discussion

In the Seniors-ENRICA-1 cohort, a well-characterized prospective Spanish cohort of older adults, higher adherence to the HBS was inversely and significantly associated with the incidence of frailty. To the best of our knowledge, this is the first study examining the association of a beverage quality index and frailty. This association was independent of numerous potential confounders, including the Mediterranean diet, which has also been consistently associated with incident frailty in this cohort [34], and in various other populations [35,36,37,38,39,40]. In addition, the most important contributor to this association was slow gait speed.

There are some previous studies estimating the association of the consumption of single beverages included in the HBS with frailty. Regarding milk, a systematic review investigated the association of dairy products intake with frailty in observational and interventional studies with community-dwelling older adults published from 2009 to 2018. A total of six studies (five observational prospective studies and one randomized clinical trial) were included. Among older adults, dairy consumption was associated with a reduced risk of frailty, especially high consumption of low-fat milk and yogurt [16]. The main result on frailty was provided from an analysis of this same cohort (Seniors-ENRICA-1). Participants consuming ≥ 7 servings/week of low-fat milk and yogurt had significantly lower incidence of frailty than those consuming < 1 serving/week. No association was found for whole milk consumption [41].

The role of milk consumption on health is controversial. Even if milk and dairy products are sources of multiple nutrients (i.e., protein, calcium, magnesium, phosphorus, potassium, zinc, selenium, vitamin A, riboflavin, vitamin B-12, and pantothenic acid), their potential benefits have come under question. There is some evidence that whole milk consumption is associated with a greater risk of all-cause, cardiovascular, and cancer mortality [42,43]. However, a recent umbrella review of meta-analyses reported that milk consumption was inversely associated with colorectal cancer risk [43]. Unfortunately, in this meta-analysis, no distinction between low-fat and whole milk was made.

Previous analyses of data from the Seniors-ENRICA-1 cohort explored the association of coffee consumption with the risk of physical function impairment, frailty, and disability in old age. Compared with non-coffee drinkers, consumption of ≥2 cups of coffee/day was associated with lower risk of impaired agility in women and in obese participants. Intake of ≥2 cups of coffee/day was also associated with reduced risk of impaired mobility in women and in participants with hypertension, while participants with diabetes who consumed ≥2 cups/day had lower risk of disability in activities of daily living [17].

In addition, tea [44] and coffee [45] consumption were associated with a lower risk of death and other health outcomes, including type 2 diabetes [44,46], coronary heart disease, and several types of cancer [43,44].

Regarding fruit juices, orange juice consumption was linked to lower frailty risk, whereas other juices were associated with a slightly higher risk (consuming ≥ 1 serving/day vs. no consumption increased the risk by about 15%) among older women in the Nurses’ Health Study [18]. Fruit juices are often high in added sugar and are ultra-processed. They have shown to be associated with some types of cancer [47], and the degree of processing of fruit-based products has also shown health implications: fresh and dried fruits appeared to have a neutral or protective effect on health, 100% fruit juices had intermediary effects, and high consumption of canned fruit and sweetened fruit juice was positively associated with a risk of all-cause mortality and type 2 diabetes, respectively [48].

In the Nurses’ Health Study, ASBs consumption has been associated with frailty in older women, although the biological mechanism remains to be elucidated. In this same study, consumption of SSBs was also associated with frailty; ≥2 servings/day vs. no SSB consumption increased the risk of frailty by about 32% [18]. Accumulated evidence shows that ASBs and SSBs are associated with an increased risk of death [49], depression [50], non-alcoholic liver disease [51], type 2 diabetes [52], hypertension, and cardiovascular disease [53]. Noteworthy, industry-sponsored research appears biased and can underestimate the adverse effects of SSBs [54].

Analyses of data from the Seniors-ENRICA-1 cohort have shown that compared with non-drinkers, the risk of frailty was significantly lower for those with higher adherence to the Mediterranean alcohol drinking pattern. This pattern was defined as moderate alcohol consumption, with a wine preference (≥80% of alcohol deriving from wine), and drinking only with meals [55]. Earlier studies in other populations had found similar results. The Women’s Health Initiative Observational Study found that moderate drinkers had a 31% lower 3-year risk of incident frailty compared to non-drinkers [56], while no association was found for heavy drinkers. However, it should be considered that in this United States sample, participants followed a pattern of alcohol consumption that is different from that in the Mediterranean drinking pattern. The Lausanne cohort 65 + study (in which wine was the predominant alcohol consumed), reported a 50% reduced risk of frailty among light-to-moderate drinkers compared with non-drinkers after 3 years of follow-up [57]. Finally, a systematic review and meta-analysis of four studies (including 44,051 participants older than 55) found that the highest alcohol consumption was associated with a lower frailty risk. Two of the included studies suggested a possible U-shaped association with the lowest risk for moderate drinkers. In this meta-analysis, heterogeneity was moderate, and there was no evidence of publication bias. Furthermore, studies on alcohol consumption are prone to bias. Limitations in these types of studies included residual confounding, the “sick quitter” effect, and survival bias [58].

Many people prefer whole milk, fruit juice, ASBs, and SSBs, which have been found to be associated with higher risk of frailty and functional decline. In general, there are no specific acceptable consumption levels for these beverages because guidelines usually recommend limiting or even avoiding this type of beverage. For example, the Dietary Guidelines for Americans 2020–2025 (DGA) [59] make a general recommendation of choosing beverages in a healthy dietary pattern that is calorie-free or that contributes beneficial nutrients, such as fat-free and low-fat milk, coffee, and tea. DGA also include, as additional strategies to lower saturated fatty acids consumption, choosing lower fat forms of foods and beverages, such as fat-free or low-fat milk instead of 2% or whole milk. Regarding fruit juice, although 100% fruit juice can be part of healthy eating patterns, it is lower than whole fruit in dietary fiber and when consumed in excess can contribute extra calories. According to DGA, if 100% fruit juice is provided, up to 4 ounces/day can fit in a healthy dietary pattern. However, juices that contain added sugars should be avoided. Many juice products, including fruit drinks, contain minimal juice content and are considered SSBs because they are primarily composed of water with added sugars. With respect to SSBs, they are considered as added sugars; beverages account for almost half (47%) of all added sugars consumed by the US population. A healthy dietary pattern limits added sugars to less than 10% of calories/day starting at age 2 and avoids beverages with added sugars for those younger than age 2. Therefore, promotion of healthy diet strategies includes choosing beverages with no added sugars. Replacing added sugars with low- and no-calorie sweeteners, as in ASBs, may reduce calorie intake in the short term and aid in weight management; nevertheless, questions remain about their effectiveness as a long-term weight management strategy. Regardless, the acceptable daily intake recommended by DGA is very low (5–50 mg/kg of body weight/day) [59].

Thus far, evidence has focused on beverages separately, and recommendations on a single beverage may not be optimal to prevent detrimental health outcomes. For example, a single isolated measure such as taxing SSBs does not necessarily improve the overall quality of beverages consumed nor diet quality as a whole [60]. In addition, a pattern may better reflect the possible synergistic or antagonistic actions of the different components than an isolated component, as it has been suggested for solid foods [61]. This is the reasoning behind our decision to combine the consumption of various beverages.

Few studies have previously examined the association of healthy beverages patterns with health outcomes. The HBI was proposed by Duffey and Davy based on data from the NHANES 2005–2010, assessing diet with 24 h recalls. They found inversely cross-sectional associations between the adherence to this index with cardio-metabolic outcomes (such as hypertension, fasting blood glucose, fasting blood insulin, and cholesterol) [25]. Another study conducted with participants with chronic kidney disease (CKD) developed a similar beverage healthy score but with a longitudinal design and found that a healthier beverage pattern was inversely associated with CKD progression and all-cause mortality [24].

Some biological mechanisms may explain our findings, considering that frailty is a condition resulting from the cumulative decline in several physiological systems. Among others, low-grade inflammation [62,63], increased oxidative stress [64], insulin resistance [65], the antioxidant properties of some beverages [66], as well as their influence in gut microbiota [67] could be considered. Thus, in experimental models, coffee administration reduced inflammatory mediators [68], while polyphenols contained in coffee induced autophagy in various tissues, a key process for the renewal of mitochondria during physical activity [69]. In addition, tea has antioxidant properties [66]. Both tea [44] and coffee [46] consumption have been associated with lower incidence of type 2 diabetes, a strong risk factor for frailty [5]. Conversely, some of the beverages included in the HBS such as fruit juices, ASBs, and SSBs may favor the development of insulin resistance and low-grade inflammation, impairing muscle glucose handling, as well as intracellular energy production, compromising efficient muscle performance [65].

Concerning gut microbiota, emerging evidence indicates that in older adults with frailty, the diversity and composition of gut microbiota are altered, which may contribute to gut permeability and dysregulation of the inflammatory response and the immune function [67]. In addition, ASBs have been shown to induce microbiota alterations favoring glucose intolerance [70], and low-fat fermented milk affects gut microbiota with its anti-inflammatory and immune-modulatory properties [71].

Our study has several strengths, including the prospective design, the collection of food consumption with a validated dietary history, adjustment for a good number of potential confounders, and a well-established definition of frailty. We acknowledge some limitations as well. The observational design precludes establishing causal inference, although the observed association between HBS and incident frailty was strong; thus, it supports an actual association. As in most studies on nutritional epidemiology, diet was self-reported, but errors in dietary assessment are expected to be non-differential; hence, it would likely bias the results toward the null. In addition, residual confounding cannot be completely ruled out.

## 5. Conclusions

In conclusion, in this prospective study in older adults, a healthy beverage pattern assessed with the HBS was associated with a reduction in half the incidence of frailty, and by almost one-third in the incidence of slow gait speed. These findings highlight the importance of beverages as critical dietary contributors to health and can help clinicians to recommend certain beverages (i.e., low fat milk or tea/coffee consumption) or avoid others (i.e., ASBs or SSBs) in order to promote healthy aging.

## Figures and Tables

**Table 1:** Scoring criteria for the Healthy Beverage Score.

Components	Minimum Score			Maximum Score
Adequacy				
Low fat milk	1 (Quartile 1)	2 (Quartile 2)	3 (Quartile 3)	4 (Quartile 4)
Tea/coffee	1 (Quartile 1)	2 (Quartile 2)	3 (Quartile 3)	4 (Quartile 4)
Moderation				
Whole milk	1 (Tertile 3 among consumers)	2 (Tertile 2 among consumers)	3 (Tertile 1 among consumers)	4 (No consumption)
Fruit Juice	1 (Any consumption)	--	--	4 (No consumption)
Artificially sweetened beverages	1 (Any consumption)	--	--	4 (No consumption)
Sugar-Sweetened beverages	1 (Any consumption)	--	--	4 (No consumption)
Alcohol	1 (No consumption or heavy drinking) ^a^	--	--	4 (Moderate drinking)
Total	7			28

^a^ Heavy drinking defined as consumption of >24 g of alcohol/day for women and >40 g of alcohol/day for men.

**Table 2:** Baseline characteristics of the Seniors-ENRICA-1 cohort participants (2008–2010) by tertiles of Healthy Beverage Score (HBS) adherence (*n* = 1900).

	HBS			
	Tertile 1 (9–19) *n* = 741	Tertile 2 (20–22) *n* = 623	Tertile 3 (23–28) *n* = 536	*p* Value
Age, mean (SD) years	69.1 (6.6)	68.5 (6.4)	68.3 (6)	0.097
Sex, % of women	49.9	60.4	44.0	<0.001
Educational level, %				0.134
Primary	50.2	56.8	52.4	
Secondary	25.8	24.1	25.4	
University	24.0	19.1	22.2	
Smoking, %				<0.001
No smoker	57.1	64.2	50.4	
Former smoker	31.7	25.0	36.0	
Current smoker	11.2	10.8	13.6	
Body mass index, %				0.941
<25 kg/m^2^	20.1	19.6	20.1	
25- < 30 kg/m^2^	50.6	49.0	49.6	
≥30 kg/m^2^	29.3	31.5	30.2	
Physical activity, %				0.079
Inactive	48.2	47.5	40.3	
Moderately inactive	30.9	33.1	34.1	
Moderately active	15.7	14.6	19.0	
Active	5.26	4.82	6.53	
Energy intake, mean (SD) Kcal/day	2089 (568)	1964 (561)	2009 (546)	<0.001
Fiber consumption, mean (SD) g/day	24 (9)	24 (8)	24 (8)	0.996
Fruit consumption, mean (SD) mL/day	321 (191)	332 (185)	321 (175)	0.510
Vegetable consumption, mean (SD) g/day	219 (146)	226 (144)	221 (141)	0.683
Mediterranean diet score (calculated with the Trichopoulou index), mean (SD)	4.51 (1.62)	4.59 (1.50)	4.50 (1.52)	0.553
Hypertriglyceridemia, %	20.2	18.6	19.0	0.731
Hypercholesterolemia, %	70.2	70.9	70.9	0.941
Hypertension, %	64.6	66.1	64.6	0.806
Number of chronic conditions ^a^, %				0.075
None	41.3	35.8	41.2	
One	41.0	41.4	40.7	
Two or more	17.7	22.8	18.1	
Number of medications, %				0.002
Three or less	48.5	56.3	48.5	
More than three	23.8	17.0	23.8	
Number of frailty components at baseline, %				
None	78.5	77.5	83.2	
One	16.3	17.2	14.2	
Two	5.1	5.3	2.6	
HBS items				
Adequacy				
Low fat milk, mean (SD) mL/day	104 (137)	184 (156)	220 (163)	<0.001
Tea/coffee, mean (SD) mL/day	89 (132)	109 (122)	173 (140)	<0.001
Moderation				
Whole milk, mean (SD) mL/day	91 (138)	25 (60)	7 (20)	<0.001
Fruit Juice, mean (SD) mL/day	65 (101)	27 (71)	8 (38)	<0.001
Artificially sweetened beverages, mean (SD) mL/day	16 (71)	7 (47)	1 (12)	<0.001
Sugar-Sweetened beverages, mean (SD) mL/day	43 (92)	11 (50)	5 (41)	<0.001
Alcohol, mean (SD) g/day	11 (22)	8 (15)	12 (13)	<0.001

^a^ Chronic conditions include coronary heart disease, stroke, chronic respiratory disease, cancer at any site, osteoarthritis/arthritis, diabetes, and depression requiring treatment.

**Table 3:** Risk of incident frailty according to tertiles of adherence to the Healthy Beverage Score in the Seniors-ENRICA-1 cohort (*n* = 1900).

Incident Frailty	Tertile 1 (Lowest) OR (95% CI)	Tertile 2 OR (95% CI)	Tertile 3 OR (95% CI)	*p* for Linear Trend
Cases, *n*	70/741	43/623	23/536	
Model 1 ^a^	1 (ref.)	0.66 (0.44,1.01)	0.51 (0.31, 0.84)	0.005
Model 2 ^b^	1 (ref.)	0.59 (0.38, 0.92)	0.51 (0.30, 0.87)	0.005
Model 3 ^c^	1 (ref.)	0.59 (0.38, 0.92)	0.52 (0.31, 0.88)	0.007

OR: odds ratio; CI: confidence interval. ^a^ Model 1 was adjusted for age (years) and sex; ^b^ Model 2 was adjusted for factors in Model 1 plus educational level (no formal education, primary and secondary or higher), smoking status (no smoke, former smoker, current smoker), BMI (<25, ≥25 and ≤30, and >30 kg/m^2^), physical activity (inactive, moderately inactive, moderately active, active), total energy intake (kcal/day, continuous), fruit consumption (g/day), vegetables consumption (g/day), fiber intake (g/day), hypertriglyceridemia (yes/no), hypercholesterolemia (yes/no), hypertension (yes/no), number of self-reported chronic conditions (0, 1 and ≥2), number of medications (0, 1–3 and >3); ^c^ Model 3 was adjusted for factors in Model 2 plus the Mediterranean diet score excluding alcohol (maximum score = 8) and excluding fruit and vegetable consumption.

**Table 4:** Risk of each frailty criterion according to tertiles of adherence to the Healthy Beverage Score in the Seniors-ENRICA-1 cohort (*n* = 1900).

	Tertile 1 (Lowest) OR (95% CI)	Tertile 2 OR (95% CI)	Tertile 3 OR (95% CI)	*p* for Linear Trend
Exhaustion				
Cases, *n*	99/741	95/623	61/536	
Model 1 ^a^	1 (ref.)	1.09 (0.80, 1.50)	0.94 (0.67, 1.34)	0.828
Model 2 ^b^	1 (ref.)	0.99 (0.71, 1.38)	0.89 (0.62, 1.29)	0.572
Model 3 ^c^	1 (ref.)	0.99 (0.71, 1.37)	0.90 (0.62, 1.29)	0.576
Weak grip strength				
Cases, *n*	268/739	212/621	176/535	
Model 1 ^a^	1 (ref.)	0.92 (0.72, 1.17)	0.97 (0.75, 1.25)	0.764
Model 2 ^b^	1 (ref.)	0.81 (0.63, 1.05)	0.89 (0.68, 1.16)	0.322
Model 3 ^c^	1 (ref.)	0.81 (0.63, 1.05)	0.89 (0.69, 1.16)	0.341
Low physical activity				
Cases, *n*	128/741	107/623	67/536	
Model 1 ^a^	1 (ref.)	0.96 (0.73, 1.28)	0.71 (0.52, 0.98)	0.048
Model 2 ^b^	1 (ref.)	0.95 (0.71, 1.28)	0.76 (0.55, 1.06)	0.120
Model 3 ^c^	1 (ref.)	0.95 (0.71, 1.28)	0.76 (0.55, 1.06)	0.118
Slow gait speed				
Cases, *n*	128/729	87/615	65/527	
Model 1 ^a^	1 (ref.)	0.80 (0.59, 1.08)	0.67 (0.49, 0.93)	0.015
Model 2 ^b^	1 (ref.)	0.79 (0.58, 1.07)	0.70 (0.50, 0.98)	0.028
Model 3 ^c^	1 (ref.)	0.79 (0.58, 1.07)	0.71 (0.51, 0.99)	0.033
Unintentional weight loss				
Cases, *n*	71/733	42/614	37/531	
Model 1 ^a^	1 (ref.)	0.65 (0.44, 0.97)	0.75 (0.49, 1.14)	0.112
Model 2 ^b^	1 (ref.)	0.63 (0.42, 0.96)	0.75 (0.49, 1.16)	0.124
Model 3 ^c^	1 (ref.)	0.63 (0.42, 0.96)	0.77 (0.50, 1.18)	0.140

^a^ Model 1 was adjusted for age (years, continuous) and sex; ^b^ Model 2 was adjusted for factors in Model 1 plus educational level (no formal education, primary and secondary or higher), smoking status (no smoke, former smoker, current smoker), BMI (<25, ≥25 and ≤30, and >30 kg/m^2^), physical activity (inactive, moderately inactive, moderately active, active), total energy intake (kcal/day, continuous), fruit consumption (g/day, continuous), vegetables consumption (g/day, continuous), fiber intake (g/day, continuous), hypertriglyceridemia (yes/no), hypercholesterolemia (yes/no), hypertension (yes/no), number of self-reported chronic conditions (0, 1 and ≥2), number of medications (0, 1–3 and >3); ^c^ Model 3 was adjusted for factors in Model 2 plus adherence to the Mediterranean diet without including alcohol (maximum score = 8) and excluding fruit and vegetable consumption.

## Data Availability

The data and the databases are available upon reasonable request to the corresponding author.

## Supplementary Materials

The following are available online at https://www.mdpi.com/article/10.3390/nu14183861/s1, Table S1: Beverages included in each item of the Healthy Beverage Index, Table S2: Risk of incident frailty according to quartiles of adherence to the Healthy Beverage Score in the Seniors-ENRICA-1 cohort (*n* = 1900), Table S3: Risk of incident frailty according to tertiles of adherence to the Healthy Beverage Score in the Seniors-ENRICA-1 cohort (*n* = 1511) among robust participants at baseline, Table S4: Risk of incident frailty according to tertiles of adherence to the Healthy Beverage Score in the Seniors-ENRICA-1 cohort (*n* = 1900) after controlling for frailty criteria at baseline, Table S5: Risk of incident frailty according to tertiles of adherence to the Healthy Beverage Score in the Seniors-ENRICA-1 cohort (*n* = 1900) considering the score without alcohol consumption and adjusting for alcohol consumption, Table S6: Risk of incident frailty according to tertiles of adherence to the Healthy Beverage Score in the Seniors-ENRICA-1 cohort (*n* = 1900) after controlling for total energy intake per ideal body weight as a proxy for body size, Table S7: Behavior of covariates and frailty risk by tertiles of the Healthy Beverage Score adherence (model 3) (*n* = 1900).
