# Supplementary material for: Adherence to a Healthy Beverage Score Is Associated with Lower Frailty Risk in Older Adults
Source: Nutrients. 2022 Sep 18;14(18):3861. doi: 10.3390/nu14183861 (PMC9501204; doi:10.3390/nu14183861)
Supplement: Supplementary file 1 [file nutrients-14-03861-s001.zip › nutrients-1877071-supplementary.pdf]

Supplementary table S1. Beverages included in each item of the Healthy Beverage Index.

**Item**	**Name of the beverages**
**Low fat milk**	Skimmed milk (0-1%), semi-skimmed milk (2%), natural skimmed fermented milk, skimmed fermented milk with fruits
**Tea/coffee**	Expresso made with and Italian coffee maker, expresso, filtered coffee, instant coffee, decaffeinated coffee made with an Italian coffee maker, decaffeinated expresso, filtered decaffeinated coffee, instant decaffeinated coffee, tea/herb tea, tea with milk, tea
**Whole milk**	Whole goat's milk, whole sheep's milk, whole dry milk, whole milk (4%), whole evaporated milk/carnation milk, whole fresh milk, natural whole fermented milk, whole fermented milk with fruits
**Fruit Juice**	Packaged exotic fruits juice, packaged lime juice, packaged apple juice, packaged orange juice, packaged pear juice, packaged pineapple juice, packaged grapefruit juice, packaged grape juice, packaged carrot juice, apricot juice/apricot nectar juice, exotic fruits juice/exotic fruits nectar juice, packaged mango nectar juice, packaged passion fruit nectar juice, packaged orange nectar juice, packaged pear nectar juice, grape juice, fruit juice, fresh orange juice, fresh grapefruit juice
**Artificially sweetened beverages**	Sparkling diet cola soft drink, lemonade
**Sugar-Sweetened beverages**	Cocoa/powdered drinking chocolate, beverage/soft drinks/sodas, sparkling lemon and lime flavored soft drink, sparkling cola soft drink, sparkling lemon flavored soft drink, still lemon flavored soft drink, sparkling orange flavored soft drink, still orange flavored soft drink, tonic water, lemon flavored slush puppy, chocolate milk, milkshakes, soya milkshake, drinking chocolate/cocoa, isotonic drink, bitter soda soft drink without alcohol, tiger nut milk, energy drink
**Alcohol**	Beer, stout bear, lager bear, cider, sweet cider, dry cider, wine, bitter soda with alcohol, sparkling wine/champagne, sangria, sparkling very dry white wine, vermouth, sweet black vermouth, white wine, sweet wine/dessert wine, sherry, rosé wine, red wine, alcoholic liqueur, alcoholic fruit liqueur, clear fruit liqueur, dark fruit liqueur, sloe gin/sloe brandy, schnapps, dry anise, brandy/cognac, baileys/Irish cream, gin, anise-flavored spirit (ouzo from Greece), rum, tequila, vodka, whisky/whiskey

Supplementary table S2. Risk of incident frailty according to quartiles of adherence to the Healthy Beverage Score in the Seniors-ENRICA-1 cohort (N=1,900).

Incident frailty	Quartile 1 (lowest) (9-18) OR (95% CI)	Quartile 2 (19-21) OR (95% CI)	Quartile 3 (22-23) OR (95% CI)	Quartile 4 (24-28) OR (95% CI)
**Cases, n**	51/558	45/535	19/342	21/465
**Model 1** [a]	1 (ref.)	0.80 (0.51, 1.25)	0.69 (0.39, 1.21)	0.52 (0.30, 0.90)
**Model 2** [b]	1 (ref.)	0.80 (0.49, 1.28)	0.69 (0.38, 1.24)	0.54 (0.31, 0.97)
**Model 3** [c]	1 (ref.)	0.78 (0.49, 1.26)	0.69 (0.38, 1.25)	0.56 (0.32, 0.39)

OR: Odds ratio; CI: Confidence interval.

[a] *Model 1 was adjusted for age (years) and sex;*

[b] *Model 2 was adjusted for factors in Model 1 plus educational level (no formal education, primary and secondary or higher), smoking status (no smoke, former smoker, current smoker), BMI (<25, ≥25 and ≤30, and >30 kg/m^2), physical activity (inactive, moderately inactive, moderately active, active), total energy intake (kcal/day, continuous), fruit consumption (g/day), vegetables consumption (g/d), fiber intake (g/d), hypertriglyceridemia, hypercholesterolemia, hypertension (yes/no), number of self-reported chronic conditions (0, 1 and ≥2), number of medications (0, 1–3 and >3);*

[c] *Model 3 was adjusted for factors in Model 2 plus the Mediterranean diet score excluding alcohol (maximum score = 8) and excluding fruit and vegetable.*

Supplementary table S3. Risk of incident frailty according to tertiles of adherence to the Healthy Beverage Score in the Seniors-ENRICA-1 cohort (N=1,511) among robust participants at baseline.

Incident frailty	Tertile 1 (lowest) OR (95% CI)	Tertile 2 OR (95% CI)	Tertile 3 (highest) OR (95% CI)	P for linear trend
**Cases, n**	32/564	19/453	12/431	
**Model 1[a]**	1 (ref.)	0.69 (0.38-1.26)	0.53 (0.26-1.05)	0.038
**Model 2[b]**	1 (ref.)	0.60 (0.32-1.14)	0.49 (0.23-1.01)	0.056
**Model 3[c]**	1 (ref.)	0.59 (0.31-1.12)	0.51 (0.25-1.06)	0.047

OR: Odds ratio; CI: Confidence interval.

[a] *Model 1 was adjusted for age (years) and sex;*

[b] *Model 2 was adjusted for factors in Model 1 plus educational level (no formal education, primary and secondary or higher), smoking status (no smoke, former smoker, current smoker), BMI (<25, ≥25 and ≤30, and >30 kg/m^2), physical activity (inactive, moderately inactive, moderately active, active), total energy intake (kcal/day, continuous), fruit consumption (g/day), vegetables consumption (g/d), fiber intake (g/d), hypertriglyceridemia, hypercholesterolemia, hypertension (yes/no), number of self-reported chronic conditions (0, 1 and ≥2), number of medications (0, 1–3 and >3);*

[c] *Model 3 was adjusted for factors in Model 2 plus the Mediterranean diet score excluding alcohol (maximum score = 8) and excluding fruit and vegetable.*

Supplementary table S4. Risk of incident frailty according to tertiles of adherence to the Healthy Beverage Score in the Seniors-ENRICA-1 cohort (N=1,900) after controlling for frailty criteria at baseline.

Incident frailty	Tertile 1 (lowest) OR (95% CI)	Tertile 2 OR (95% CI)	Tertile 3 (highest) OR (95% CI)	P for linear trend
**Cases, n**	70/741	43/623	23/536	
**Model 1[a]**	1 (ref.)	0.66 (0.44-1.01)	0.51 (0.31-0.84)	0.005
**Model 2[b]**	1 (ref.)	0.59 (0.38-0.92)	0.51 (0.30-0.87)	0.005
**Model 3[c]**	1 (ref.)	0.60 (0.38-0.95)	0.58 (0.34-0.99)	0.021

OR: Odds ratio; CI: Confidence interval.

[a] *Model 1 was adjusted for age (years) and sex;*

[b] *Model 2 was adjusted for factors in Model 1 plus educational level (no formal education, primary and secondary or higher), smoking status (no smoke, former smoker, current smoker), BMI (<25, ≥25 and ≤30, and >30 kg/m²), physical activity (inactive, moderately inactive, moderately active, active), total energy intake (kcal/day, continuous), fruit consumption (g/day), vegetables consumption (g/d), fiber intake (g/d), hypertriglyceridemia, hypercholesterolemia, hypertension (yes/no), number of self-reported chronic conditions (0, 1 and ≥2), number of medications (0, 1–3 and >3);*

[c] *Model 3 was adjusted for factors in Model 2 plus the Mediterranean diet score excluding alcohol (maximum score = 8) and excluding fruit and vegetable.*

Supplementary table S5. Risk of incident frailty according to tertiles of adherence to the Healthy Beverage Score in the Seniors-ENRICA-1 cohort (N=1,900) considering the score without alcohol consumption and adjusting for alcohol consumption.

Incident frailty	Tertile 1 (lowest) OR (95% CI)	Tertile 2 OR (95% CI)	Tertile 3 (highest) OR (95% CI)	P for linear trend
**Cases, n**	75/726	29/527	32/511	
**Model 1[a]**	1 (ref.)	0.56 (0.35-0.89)	0.64 (0.41-1.01)	0.027
**Model 2[b]**	1 (ref.)	0.46 (0.28-0.76)	0.59 (0.37-0.94)	0.011
**Model 3[c]**	1 (ref.)	0.46 (0.28-0.77)	0.63 (0.39-1.02)	0.025

OR: Odds ratio; CI: Confidence interval.

[a] *Model 1 was adjusted for age (years) and sex;*

[b] *Model 2 was adjusted for factors in Model 1 plus educational level (no formal education, primary and secondary or higher), smoking status (no smoke, former smoker, current smoker), alcohol consumption, BMI (<25, ≥25 and ≤30, and >30 kg/m²), physical activity (inactive, moderately inactive, moderately active, active), total energy intake (kcal/day, continuous), fruit consumption (g/day), vegetables consumption (g/d), fiber intake (g/d), hypertriglyceridemia, hypercholesterolemia, hypertension (yes/no), number of self-reported chronic conditions (0, 1 and ≥2), number of medications (0, 1–3 and >3);*

[c] *Model 3 was adjusted for factors in Model 2 plus the Mediterranean diet score excluding alcohol (maximum score = 8) and excluding fruit and vegetable.*

Supplementary table S6. Risk of incident frailty according to tertiles of adherence to the Healthy Beverage Score in the Seniors-ENRICA-1 cohort (N = 1,900) after controlling for total energy intake per ideal body weight as a proxy for body size.

Incident frailty	Tertile 1 OR (95% CI)	Tertile 2 OR (95% CI)	Tertile 3 OR (95% CI)	P for linear trend
**Cases, n**	70/741	43/623	23/536	
**Model 4** [a]	1 (ref.)	0.57 (0.34-0.96)	0.52 (0.28-0.95)	0.016
**Model 5** [b]	1 (ref.)	0.58 (0.34-0.97)	0.53 (0.29-0.98)	0.020

OR: Odds ratio; CI: Confidence interval.

[a] *Model 4 was adjusted for age (years, continuous), sex, educational level (no formal education, primary and secondary or higher), smoking status (no smoke, former smoker, current smoker), BMI (<25, ≥25 and ≤30, and >30 kg/m²), physical activity (inactive, moderately inactive, moderately active, active), total energy intake per ideal body weight (kcal/kg of ideal body weight/day, continuous), fruit consumption (g/day, continuous), vegetables consumption (g/d, continuous), fiber intake (g/d, continuous), hypertriglyceridemia (yes/no), hypercholesterolemia (yes/no), hypertension (yes/no), number of self-reported chronic conditions (0, 1 and ≥2), number of medications (0, 1–3 and >3);*

[b] *Model 5 was adjusted for factors in Model 4 plus adherence to the Mediterranean diet without including alcohol (maximum score = 8) and excluding fruit, and vegetable consumption.*

Supplementary table S7. Behavior of covariates and frailty risk by tertiles of the Healthy Beverage Score adherence (model 3) (N=1,900).

	Frailty risk OR (95% IC)	P value
Age (continuous)	1.12 (1.09-1.15)	<0.001
Sex (women)	2.87 (1.61-5.12)	<0.001
Educational level		
Primary	1 (ref.)	
Secondary	0.43 (0.24-0.77)	0.005
University	0.45 (0.24-0.85)	0.014
Smoking		
No smoker	1 (ref.)	
Former smoker	1.70 (0.98-2.97)	0.061
Current smoker	0.93 (0.39-2.23)	0.877
BMI		
<25 kg/m^2	1 (ref.)	
25-<30 kg/m^2	1.27 (0.68-2.40)	0.455
≥30 kg/m^2	2.14 (1.12-4.07)	0.021
Physical activity		
Inactive	1 (ref.)	
Moderately inactive	0.49 (0.30-0.79)	0.003
Moderately active	0.47 (0.24-0.93)	0.029
Active	0.51 (0.15-1.75)	0.284
Energy intake, mean (SD) (continuous)	1.00 (1.00-1.00)	0.778
Fiber consumption (continuous)	0.97 (0.94-1.01)	0.166
Mediterranean diet score (continuous)	0.94 (0.82-1.08)	0.374
Hypertriglyceridemia	1.73 (1.11-2.70)	0.015
Hypercholesterolemia	0.79 (0.51-1.21)	0.279
Hypertension	1.84 (0.54-1.31)	0.445
Number of chronic conditions		
None	1 (ref.)	
One	2.16 (1.23-3.80)	0.007
Two or more	2.66 (1.42-4.96)	0.002
Number of medications		
Three or less	1.02 (0.58-1.79)	0.06
More than three	1.49 (0.79-2.81)	0.215
